# Supplementary figures and images for: Analysis of Promoter-Associated Chromatin Interactions Reveals Biologically Relevant Candidate Target Genes at Endometrial Cancer Risk Loci
Source: Cancers (Basel). 2019 Sep 26;11(10):1440. doi: 10.3390/cancers11101440 (PMC6826789; doi:10.3390/cancers11101440)

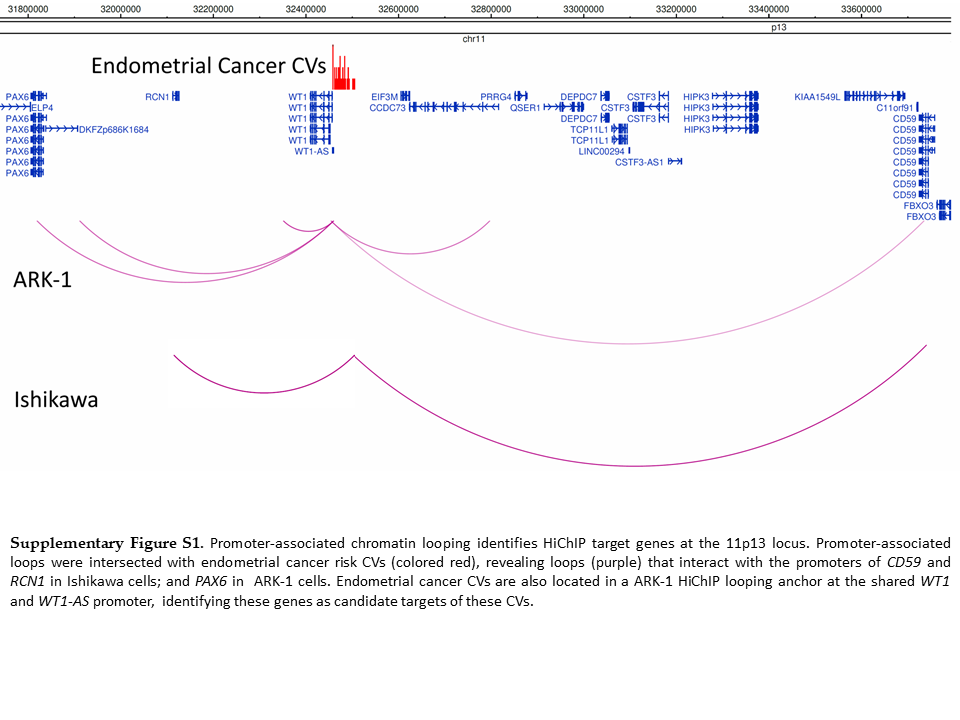

Supplement: Supplementary file 1 [file cancers-11-01440-s001.zip › Supp Figure S1.tif]

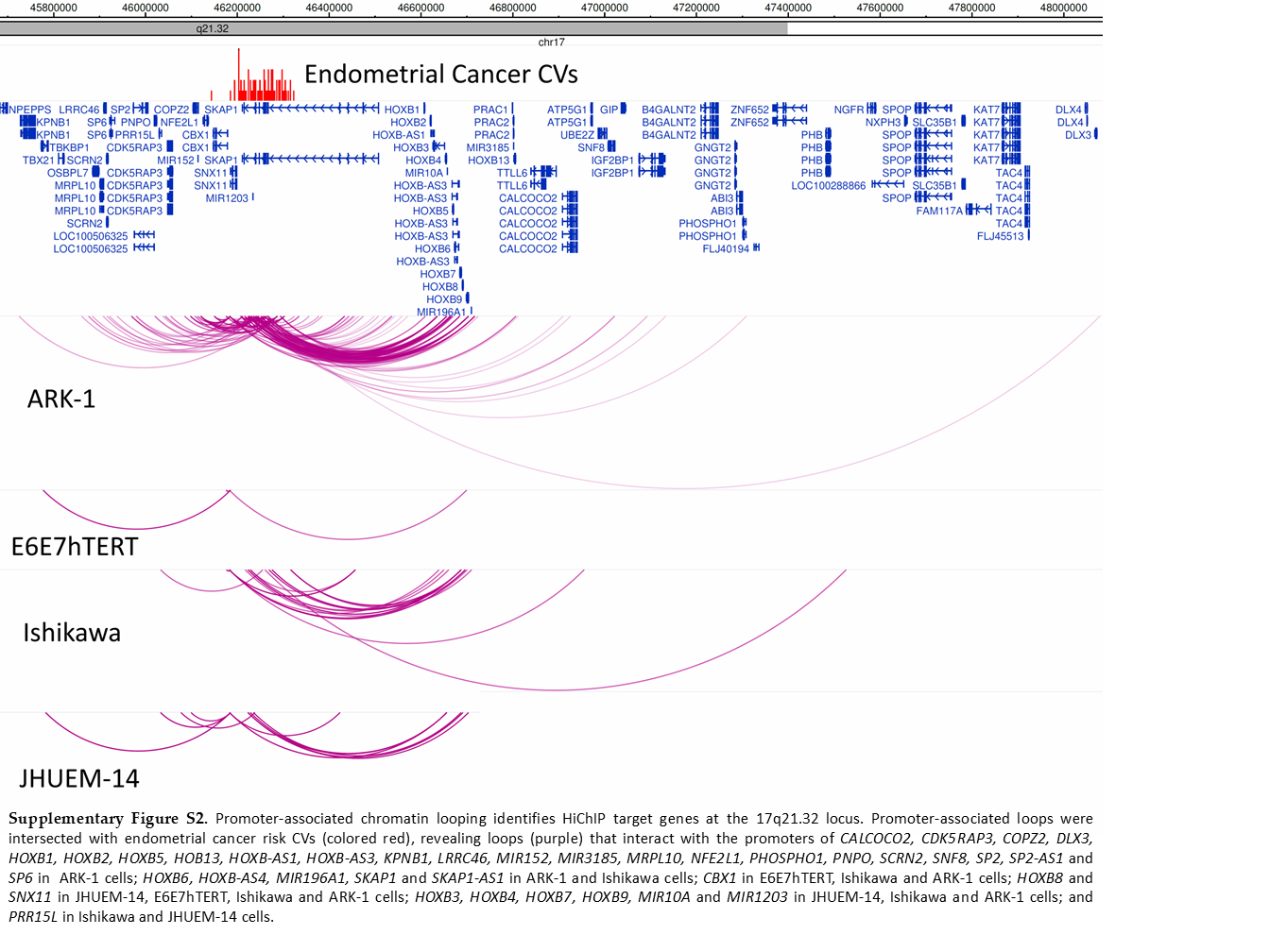

Supplement: Supplementary file 1 [file cancers-11-01440-s001.zip › Supp Figure S2.tif]

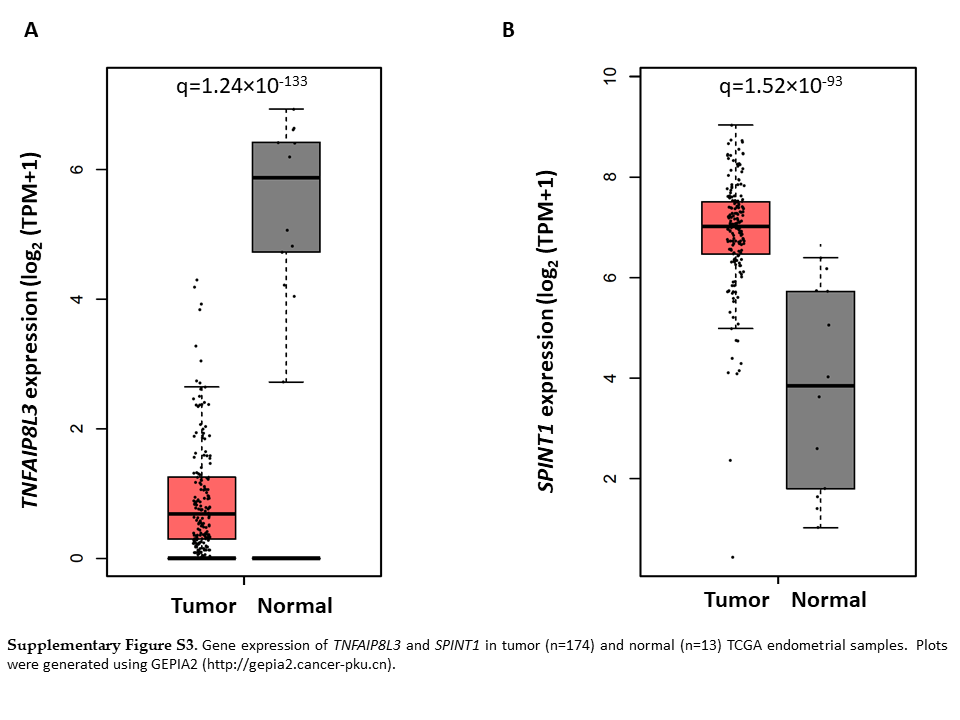

Supplement: Supplementary file 1 [file cancers-11-01440-s001.zip › Supp Figure S3.tif]
